# An age-adapted plyometric exercise program improves dynamic strength, jump performance and functional capacity in older men either similarly or more than traditional resistance training

**DOI:** 10.1371/journal.pone.0237921

**Published:** 2020-08-25

**Authors:** Evelien Van Roie, Simon Walker, Stijn Van Driessche, Tijs Delabastita, Benedicte Vanwanseele, Christophe Delecluse

**Affiliations:** 1 Physical Activity, Sports and Health Research Group, Department of Movement Sciences, KU Leuven, Leuven, Belgium; 2 Neuromuscular Research Center, Faculty of Sport and Health Sciences, University of Jyväskylä, Jyväskylä, Finland; 3 Human Movement Biomechanics Research Group, Department of Movement Sciences, KU Leuven, Leuven, Belgium; Universita degli Studi di Roma 'Foro Italico', ITALY

## Abstract

Power declines at a greater rate during ageing and is more relevant for functional deterioration than either loss of maximum strength or muscle mass. Human movement typically consists of stretch-shortening cycle action. Therefore, plyometric exercises, using an eccentric phase quickly followed by a concentric phase to optimize power production, should resemble daily function more than traditional resistance training, which primarily builds force production capacity in general. However, it is unclear whether older adults can sustain such high-impact training. This study compared the effects of plyometric exercise (PLYO) on power, force production, jump and functional performance to traditional resistance training (RT) and walking (WALK) in older men. Importantly, feasibility was investigated. Forty men (69.5 ± 3.9 years) were randomized to 12-weeks of PLYO (N = 14), RT (N = 12) or WALK (N = 14). Leg press one-repetition maximum (1-RM), leg-extensor isometric maximum voluntary contraction (MVC) and rate of force development (RFD), jump and functional performance were evaluated pre- and post-intervention. One subject in RT (low back pain) and three in PLYO (2 muscle strains, 1 knee pain) dropped out. Adherence to (91.2 ± 4.4%) and acceptability of (≥ 7/10) PLYO was high. 1-RM improved more in RT (25.0 ± 10.0%) and PLYO (23.0 ± 13.6%) than in WALK (2.9 ± 13.7%) (p < 0.001). PLYO improved more on jump height, jump power, contraction time of jumps and stair climbing performance compared to WALK and/or RT (p < 0.05). MVC improved in RT only (p = 0.028) and RFD did not improve (p > 0.05). To conclude, PLYO is beneficial over RT for improving power, jump and stair climbing performance without compromising gains in strength. This form of training seems feasible, but contains an inherent higher risk for injuries, which should be taken into account when designing programs for older adults.

## Introduction

During the ageing process, muscle mass, muscle force and power production decline progressively [1–3]. This decline is more pronounced in power and rapid force production than in maximal force or muscle mass [3, 4]. In addition, the ability to generate a high amount of power enables individuals to perform better on everyday activities, such as rising from a chair and climbing stairs [5]. In reactive motor tasks, such as balance recovery following sudden perturbations, muscle force needs to be generated in short time frames [6]. Consequently, reduced lower-limb power and slowing of force production have been proposed as important predictors of age-related deterioration in functional performance and should be targeted in exercise programs for older adults.

Resistance exercise has been widely recognized as an effective strategy to improve muscle mass, muscle strength and functional performance in older adults. The majority of interventions prescribed slow-speed resistance exercise with 1–4 sets of about 8–15 repetitions at moderate to high loads for at least 2x/week, in line with previous international recommendations [7]. Although the benefits are well documented, limited improvements in power and rate of force production have been reported when exercises are typically performed at slow and controlled speeds [8]. As mentioned above and supported by recent insights and exercise guidelines, there are important arguments to justify the inclusion of explosive type of resistance exercise in older adults [9, 10]. Machine-based resistance exercise performed with an explosive concentric phase followed by a controlled, slower eccentric phase is feasible, even in institutionalized elderly [11]. This type of explosive resistance exercise has shown greater effects on functional performance than traditional slow-speed resistance exercise [12, 13].

However, human locomotion rarely involves pure concentric movements, but consists of rapidly coupled eccentric-concentric multi-joint muscle actions, known as stretch-shortening cycle (SSC) activities. Fast eccentric-concentric transition in a SSC movement facilitates subsequent power generation through storage and reutilization of elastic energy [14]. Therefore, multi-joint SSC movements represent a mechanism behind optimal power production. This is an important argument for the use of plyometric training, which specifically targets multi-joint SSC, in older adults, especially considering that the utilization of elastic energy becomes gradually impaired due to neural and structural changes in aged muscles [15, 16].

Plyometric training elicits numerous positive changes in neural and musculoskeletal systems, muscle function and performance of healthy individuals (for a review, see [17]). However, this training modality has primarily been used in athletes and/or young adults, while limited research exists on plyometric training for older adults [18]. In addition, knowledge on the potential adverse events of such high-impact training is lacking, as papers often fail to comment on feasibility and injuries [19].

Therefore, we designed a 12-week multi-joint plyometric exercise program (PLYO) for older men. This program was age-adapted to maximize feasibility by slowly progressing from slow speed exercises over submaximal to maximal jumps, using only low-intensity drills, allowing using wall bars for support and introducing short breaks between every jump. Its effects on muscle strength, muscle power, jump performance and functional capacity were compared to a traditional resistance training program (RT) and a walking program (WALK). The walking group was considered a control group, as the exercise was not likely to improve muscle strength or power, but did consist of low-load eccentric-concentric lower-limb movements that might already affect functional performance. As a secondary outcome, the feasibility of the plyometric exercise program was investigated. We hypothesized that PLYO would improve more on muscle power, jump performance and functional performance than RT and WALK and that the program would be feasible in healthy community-dwelling older men.

## Methods

### Trial design

This randomized trial was designed as a parallel-group study, with three different exercise interventions. The intervention duration was 12 weeks. Outcome measurements were obtained at baseline (pre-intervention, from February to March 2018) and within one week after the last exercise session (post-intervention, from May to June 2018). The study was approved by the Human Ethics Committee Research UZ/KU Leuven in accordance with the declaration of Helsinki. All subjects provided written informed consent.

### Subjects

**Fig 1:**
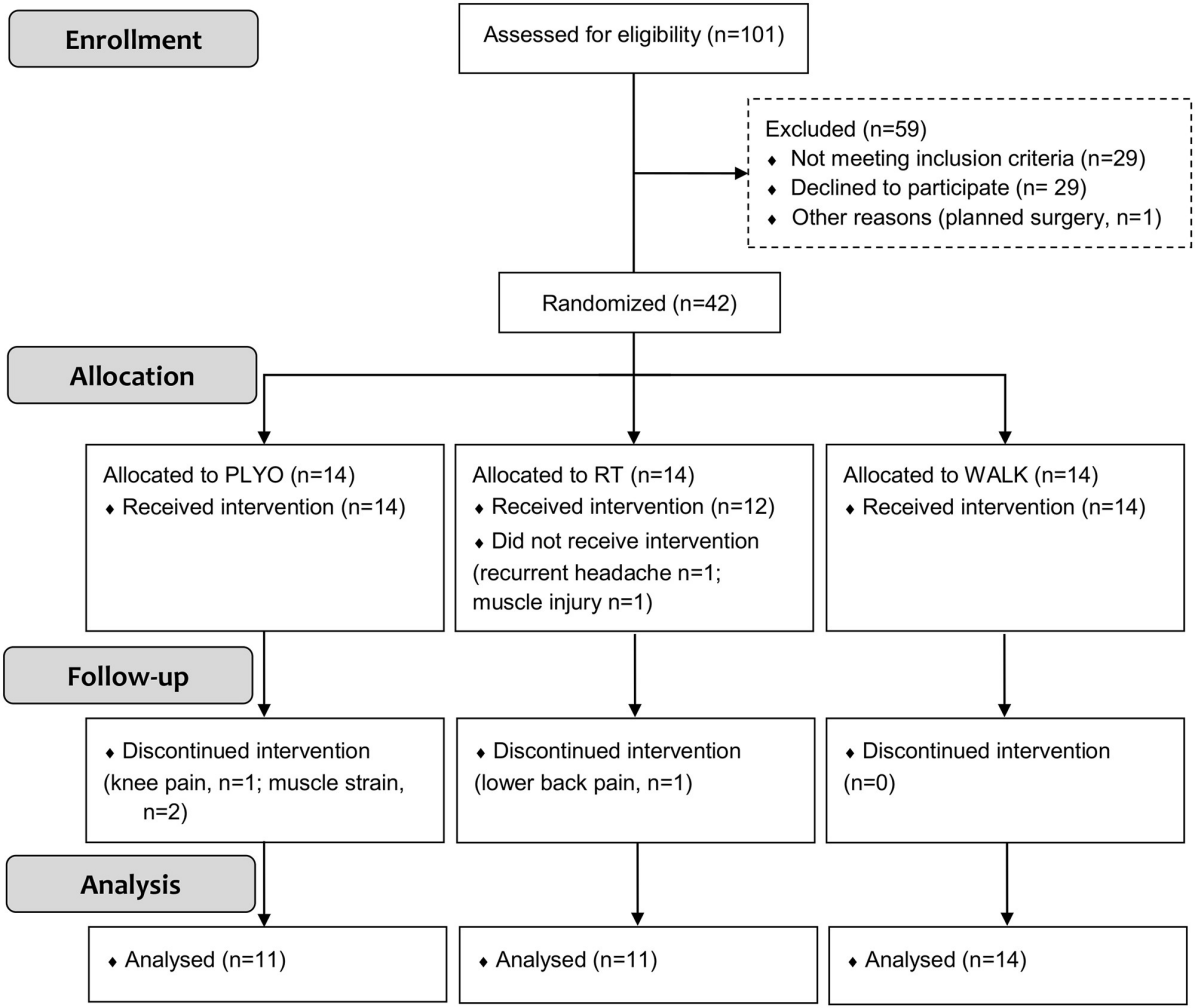
Flowchart of the study. PLYO = plyometric training; RT = resistance training; WALK = walking.

Community-dwelling older men aged 65 to 80 years were recruited through advertisements in local newspapers. Exclusion criteria were unstable cardiovascular disease, neurological disorders, cognitive malfunctioning, severe knee or hip problems, previous rupture of the Achilles tendon and systematic engagement in (resistance) exercise in the 12 months prior to participation. In total, 42 men (age: 69.4 ± 3.8 years, body mass: 82.7 ± 10.5 kg, body height: 174.8 ± 6.4 cm, BMI: 27.1 ± 3.2 kg/m^2^) were found eligible and agreed to participate in the study (Fig 1).

### Randomization

Subjects were randomly assigned to one of three intervention conditions by block randomization (block size of 3): traditional resistance training (RT, n = 14), plyometric exercise (PLYO, n = 14) or walking (WALK, n = 14). Allocation ratio was 1:1:1. In RT, two subjects were not able to perform baseline measurements because of recurrent headache or muscle injury so they were excluded from the study (Fig 1).

### Exercise protocols

#### Resistance training protocol

Subjects exercised three times weekly on non-consecutive days over a period of 12 weeks (total of 36 sessions). Exercise sessions were performed in small groups of maximum four subjects and were supervised by at least one expert. Session duration was about 35 minutes. After a standard 15-minute warm-up on a cycle ergometer (Technogym, Bike Excite) at self-selected resistance and 70–80 revolutions per minute, three exercises for the lower-limb muscles were performed: the bilateral leg press and straight-legged calf raises (both on the plate-loaded linear leg press, Life Fitness Signature Series) and leg extension (Life Fitness Optima Series). These exercises were chosen because they train the muscles responsible for triple extension in the lower-limb (i.e. knee extensors, hip extensors and plantar flexors), a multi-joint movement that is crucial in daily life activities such as walking and stair climbing. Training variables and progression are shown in Table 1. Subjects were instructed to perform the last set to concentric failure. When they were able to perform more repetitions than the prescribed training zone, the load was increased in the next exercise session. The rest period between sets and exercises was one minute and at least two minutes, respectively. At the end of the exercise session, basic static stretching exercises were performed for the trained muscle groups.

**Table 1:** Training variables for the resistance training (RT) and plyometric (PLYO) training program.

	Sets	Repetitions	External load	Break between repetitions	Inter-set rest	Performance
RT (leg press, straight-legged calf raise, leg extension)						
Week 1–4	2	12–15	12-15RM	/	1 min.	3s ecc– 3 sec conc
Week 5–8	3	10–12	10-12RM	/	1 min.	3s ecc– 3 sec conc
Week 9–12	4	8–10	8-10RM	/	1 min.	3s ecc– 3 sec conc
PLYO (forward step-up, lateral step-up, countermovement jump)						
Week 1–3	2	15–20	BM*	/	1 min.	Regular speed, no jump
Week 4–6	3	8–12	BM	5s	1 min.	Fast ecc–submax. jump
Week 7–9	4	6–8	BM	5s	1 min.	Fast ecc–max. jump
Week 10–12	4	6–8	BM	/	1 min.	Fast ecc–max. jump

* Progression of intensity during week 1–3 in PLYO was based on subjects’ feelings of muscle fatigue: forward and lateral step-up: increase in step height (to maximum 40 cm); squat: adding weight vest of 5 to 10 kg.

BM = body mass; RM = repetition maximum (last set performed to concentric failure in RT); ecc = eccentric.

#### Plyometric exercise protocol

**Fig 2:**
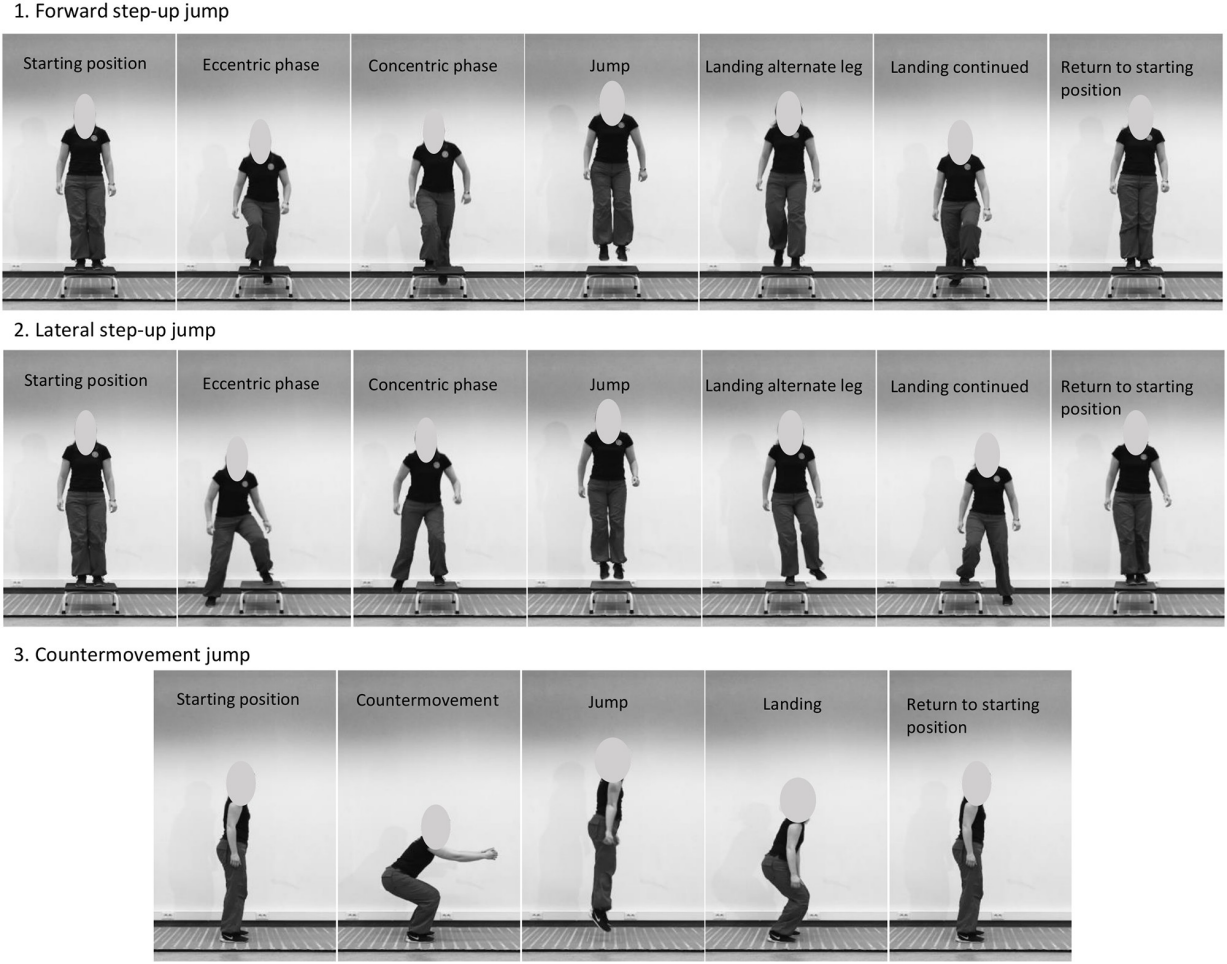
Visual representation of the three plyometric exercises from week 4 to 6.

Similar to RT, training sessions (35-minute duration) in PLYO were performed three times weekly on non-consecutive days for 12 weeks in small groups of maximum four subjects and supervised by at least one expert. The warm-up consisted of 10-minutes of cycling, followed by plyometric warm-up exercises: 4 x 10m high knee skips, 4 x 10m sideways skips and 8 consecutive hops with short contact times. The core program similarly consisted of three exercises, using the same muscle groups (triple extension) as in RT: two unilateral exercises, i.e. forward step-up (jump) and lateral step-up (jump), and one bilateral exercise, i.e. countermovement jump. Exercise intensity progressively increased every three weeks (see Table 1). The rest period between sets and exercises was one minute and at least two minutes, respectively, in all training phases. From week 1–3, exercises were performed at regular speed without jumping, i.e. either stepping up on a box (20-30-40 cm) in forward or lateral direction or performing a squat (body mass with or without weight vest) with bouncing movement at the lowest point. Weight vests (5–10 kg) were only used for the squat exercise prior to progressing to jumping. From week 4–6, all exercises progressed to submaximal jumping with a short eccentric phase before all jumps (i.e. SSC action) (Fig 2) and weight vests were no longer used as the squat exercise progressed to a countermovement jump exercise. Subjects were instructed to have a short 5s-break between the repetitions to ensure proper performance and to avoid fatigue. From week 7–9, subjects were instructed to jump as explosively and as high as possible in every jump, with 5s-breaks between the repetitions. From week 10–12, both step-up jumps were performed consecutively (no breaks between repetitions, short contact times) and as high as possible. The countermovement jumps were preceded by a two pre-hops, the second one with countermovement to load both ankle and hip/knee joints. In all exercise phases, subjects were allowed to use wall bars for support if needed and were instructed to stop the exercise when feeling unable to perform maximally. At the end of the exercise session, basic static stretching exercises were performed for the trained muscle groups.

### Walking protocol

The walking program was adapted from a 10-week progressive individualized walking program, described in detail in previous work [20], by adding two additional weekly schedules to complete a 12-week program (see Table 2). Briefly, subjects were assigned to a starting level of the walking program based on the results of a 6-minute walk test at baseline. Each walking schedule prescribed walks of a certain number of steps on 3–7 days a week, depending on the starting level. Training volume was progressively increased weekly to a maximum of walks of 10000 steps performed three times weekly. Subjects received their personalized walking schedule and a pedometer. They were instructed to walk at a moderate and comfortable pace that increased breathing and heart rate without restricting the ability to talk. Subjects were allowed to perform their walks at home, but were encouraged to engage in group walks that were provided three times weekly at the training facility. All subjects were asked to document their walk sessions, including the amount of steps performed, in a diary. Diaries were reviewed by the research team when subjects joined the on-site group walks (on average once per week) or at least once every two weeks through contact via e-mail or telephone.

**Table 2:** Walking program (prescribed number of steps per day).

level	N	day 1	day 2	day 3	day 4	day 5	day 6	day 7	sessions per week	volume per week
1		1000**	1000**	1000**	1000**	1000**	1000**	1000**	7	7000
2		1000*	1000*	1000*	1000*	1000*	1000*	1000*	7	7000
3		1000	1000	1000	1000	1000	1000	1000	7	7000
4		1500	1000	1500	1000	1500	1000	1500	7	9000
5		1500	1500	1500	1500	1500	1500	1500	7	10500
6		2000	1500	2000	1500	2000	1500	2000	7	12500
7	4	2000	2000	2000	2000	2000	2000	2000	7	14000
8		2500	2000	2500		2500	2000	2500	6	14000
9	1	3000	2000	3000		3000	2000	3000	6	16000
10	2	3000	3000	3000		3000	3000	3000	6	18000
11	2	4000		3000		4000		3000	4	14000
12	5	4000		4000		4000		4000	4	16000
13		4000		4000		4000		4000	4	16000
14		6000		4000		6000		4000	4	20000
15		6000		4000		6000		4000	4	20000
16		8000		4000		6000		4000	4	22000
17		8000		4000		8000		4000	4	24000
18		8000		8000			8000		3	24000
19		8000		6000			10000		3	24000
20		10000		5000			10000		3	25000
21		10000		7000			10000		3	27000
22		10000		10000			10000		3	30000
23		10000		10000			10000		3	30000

*1 resting break allowed,

**2 resting breaks allowed.

N represents the number of subjects with the respective level as starting level of the program. Every week, subjects increased one level until the 12-week intervention was finished.

### Outcome measurements

#### Feasibility

Feasibility of the exercise protocols were assessed by the following criteria: recruitment rate, exercise session adherence, number of drop-outs, acceptability and adverse events [21]. Recruitment rate was calculated as the number of study subjects divided by the total number of individuals that showed an initial interest in study participation. Adherence rate was calculated as the number of training sessions performed divided by the recommended training frequency (3x/week for 12 weeks, 36 sessions for PLYO and RT; 3-6x/week for 12 weeks, 41–56 sessions for WALK). Given that subjects in WALK could potentially perform more sessions than recommended, which was not the case for PLYO and RT, we additionally corrected the adherence rate in WALK. More specifically, the corrected adherence rate in WALK was calculated by excluding any exercise sessions above the weekly prescribed number of exercise sessions. For example, if a subject performed 5 exercise sessions instead of 4 in a certain week, adherence was corrected to 100% instead of 125% for that week. The number of drop-outs was recorded, including the time of and the reason for drop-out. All subjects were asked to report any adverse events during the intervention.

Acceptability was evaluated through a short questionnaire completed 2-weekly in RT and PLYO only. The questionnaire consisted of 5 questions answered on a 11-point Likert scale (ranging from 0 = ‘not at all…’ to 10 = ‘very…’): (1) How much did you enjoy the exercises while doing them? (2) How proud are you that you were able to complete these exercises? (3) How confident are you that you will be able to complete these exercises in the next training session? (4) How motivated are you to complete these exercises in the next training session? (5) How feasible do you think that these exercises are for people of your age? The first four questions were employed previously in similar populations [22, 23], while question 5 was added. In week 12, a sixth question was added to the questionnaire: (6) How likely is it that you will engage in similar exercise programs after the end of the intervention? The questionnaire in week 12 was also provided to the subjects of WALK. Internal consistency of the 5-item questionnaire was good, with a Cronbach’s α coefficient of 0.79. Therefore, we calculated the mean of the 5 items into one global scale representing acceptability of the intervention program.

#### Leg press one-repetition maximum

Leg press one-repetition maximum (1-RM) was assessed on the plate loaded linear leg press device (Life Fitness Signature Series). In accordance with previous research [22], the assessment started with a standardized warm-up of 8 repetitions at 50% of the estimated 1-RM, followed by 5 repetitions at 70% of the estimated 1-RM. After this warm-up, single lifts with progressively heavier loads were performed until failure. To standardize, these lifts were performed as concentric lifts only, starting in a knee and hip joint angle of 90° and 65° respectively (full extension = 180°). Rest periods between warm-up sets and between single attempts were 1 to 5 minutes. The heaviest successful lift (in kg) was determined as 1-RM.

#### Force production and jump performance

**Fig 3:**
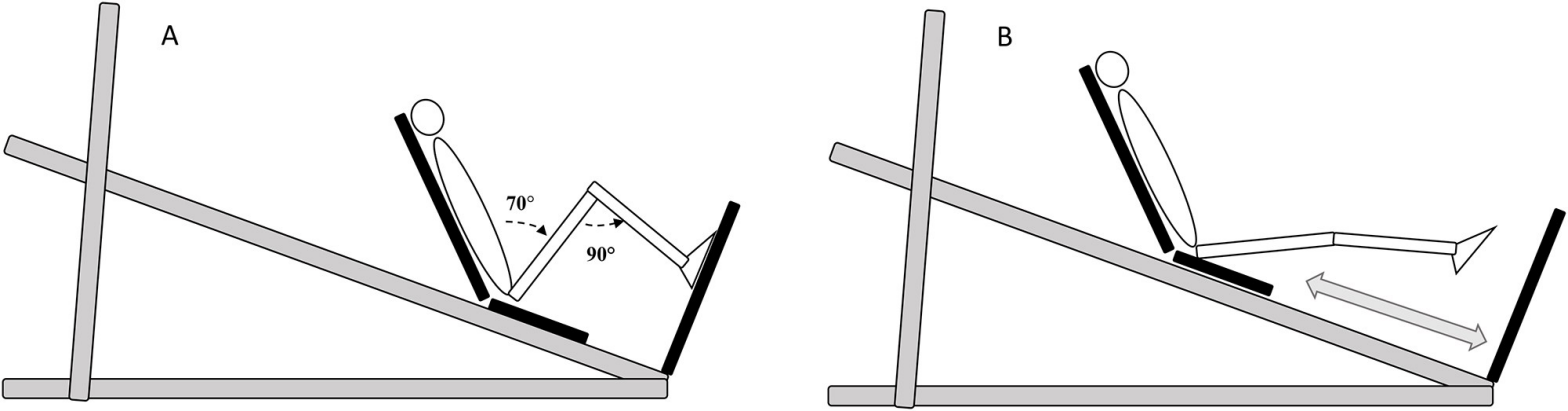
Sledge apparatus with a force platform built in perpendicular to the jumping direction and a speed sensor attached to the seat of the sledge. Position A represents the lowest position in the isometric tests and in the squat jumps, position B represents the flight phase during jumps.

A sledge apparatus was used to assess leg-extensor force production and jump performance [24]. The inclination of the sledge was 20° to horizontal and the seat was inclined backwards (130°). A force platform was built in perpendicular to the jumping direction (Fig 3) and a velocity sensor was attached to the seat of the sledge. The force platform consisted of four S style load cells (YZC-516, capacity of 300kg each) that were attached to a custom-built platform. Prior to attachment to the platform, each of the load cells was calibrated with a weight of 155kg. In addition, the platform was calibrated by measuring the unloaded condition prior to each test. The velocity sensor consists of a permanent magnet DC motor riding along with the seat. A wheel running on a rail next to the seat of the sledge drives the motor shaft. That way the motor acts as a generator, producing a current linear with the rotational velocity. Calibration was done by measuring the current while running the wheel at different velocities. Rotational velocity was measured with an electronic pulse counter (HP53131A) counting the pulses of a temporarily added pulse generator on the shaft. The current was measured with a high precision multimeter (HP34405A). Linear regression analysis showed a R^2^ of 0.9998 between current and velocity. The shoulders and hips were stabilized with a 4-point seatbelt. Subjects wore standard flat non-cushioning shoes to minimize the cushioning effect during explosive movements.

The test protocol consisted of explosive isometric voluntary contractions, squat jumps (SJ), countermovement jumps (CMJ), and drop jumps (DJ). Prior to all tests, 2 to 4 practice trials were allowed. All tests were performed three times. All data were relayed to a pc via an AD converter (Micro 1401, Cambridge Electronic 180 Design, UK) and recorded using Signal 4.03 software (Cambridge Electronic Design, UK). Data (time, force and velocity) were sampled at 1000Hz and force data were filtered using a fourth-order low-pass Butterworth filter with a 70Hz cut-off frequency. The best trial (maximal force, maximal rate of force development or highest jump) was used for further analyses.

Explosive isometric multi-joint leg-extensor contractions were performed unilaterally with the right leg while the sledge was locked in position. The knee joint angle was set at 90° and the hip angle at 70°. The point of force application was aligned with the head of the fifth metatarsal. Subjects were instructed to push as fast and as hard as possible and maintain their maximum force for approximately 3s. The start of the force-time curve was defined as the first point where the force exceeded body weight by 3% and where the increase in force after 50ms was at least 50N (to ensure the start of a slope). Maximal force (N) was defined as the highest mean 500 ms epoch (rolling average) over the force-time curve. The rate of force development (N/s) was defined as the linear slope of the force-time curve and was measured from the onset of movement until 100 ms (RFD0-100).

In the bilateral SJ, subjects jumped with the sledge as high as possible from a squatting position (90° knee flexion and 70° hip angle, same position as isometric tests) without countermovement by performing a fast upward movement. If the test leader visually detected a countermovement, the jump was repeated. The start of the jump was defined as the first point where the force exceeded body weight by 3% and the increase in velocity exceeded 0.02 m/s after 300 ms. Any jump preceded by a countermovement, defined as a drop in velocity of at least -0.02 m/s in the 400 ms preceding the start of the jump, was deleted from the analyses.

In the bilateral CMJ, subjects jumped as high as possible by starting from an extended knee joint position and performing a fast downward movement to about 90° knee flexion and 80° hip flexion, immediately followed by a fast upward movement. The start of the jump was defined as the first point where the force dropped below body weight by 3% and the decrease in velocity dropped below -0.02 m/s after 300 ms.

Bilateral drop jumps were performed as three maximal consecutive jumps. Feet were placed lower on the force plate to allow for continuous jumps on the forefoot (hip joint angle of 130° in extended position). Subjects were asked to reverse the downward velocity as soon as possible after landing on the force platform into an upward one. The second and third jump were used as maximal drop jump performances. The start of the second or third jump was defined as the point of landing (time point before force ≥ 10N) after the first or second flight phase.

For the jump tests, instantaneous power was calculated as the product of force and velocity. Instantaneous position of the chair was derived from the velocity signal. The following parameters were calculated: (1) jump height (m), i.e. the difference between the maximal value of the position-time curve and the position at take-off (point before force ≤ 10N); (2) contraction time (s), i.e. the time from the start of the jump until take-off; (3) eccentric time (s) (for CMJ and DJ), i.e. the time from the start of the jump until velocity > 0; (4) concentric time (s) (for CMJ and DJ), i.e. the time from the end of the eccentric phase until take-off; (5) reactive strength index (RSI, mm/s, for DJ), i.e. jump height (in mm) divided by contraction time; (6) concentric peak power (watt) (Ppeak), i.e. the highest value of the power-time curve in the concentric phase; and (7) concentric rate of power development (RPD, watt/s), i.e. the linear slope of the power-time curve from start until peak in the concentric phase.

All settings of the sledge and subjects’ positioning were identical at baseline and post intervention. Comparison of familiarization and baseline measurements showed that coefficients of variation (CV, in %) ranged from 5.7% to 18.9% for all parameters measured with the sledge apparatus. Additional reliability values are reported in S1 and S2 Tables. Acceptable reliability was determined as an intraclass correlation coefficient (ICC) > 0.60 [25] and a CV < 15% [26].

#### Functional performance

Functional performance was assessed by a test battery, consisting of a 6-minute walk test (6MWT), a 10m fast walk, a 5-repetition sit-to-stand (5xSTS) test and a 6-step stair ascent (SA) test.

In the 6MWT, subjects were asked to walk a 20m course (back and forth) at a fast but comfortable pace, and the total distance covered (m) was noted. In the 10m fast walk test, subjects were asked to walk as fast as possible. Time (s) was registered through timing gates (Racetime2 Light Radio, Microgate, IT). During the 5xSTS and SA test, data were collected by means of 3D accelerometry positioned at the lower back (DynaPort MoveTest, McRoberts, The Hague, NL). Sampling rate was 100Hz and data were analyzed using commercially available software (DynaPort MoveTest, McRoberts, The Hague, NL). In the 5xSTS, subjects were instructed to perform five sit-to-stand cycles as fast as possible with the arms crossed over the chest. Total STS duration (from start until the fifth standing position) in seconds was calculated from the accelerometer data [27]. Mean power (watt) was calculated for each single sit-to-stand transition and the highest mean power output was used in the analyses. In the SA test, subjects ascended a flight of 6 stairs as fast as possible without using the handrail. Total SA duration (s) and mean power (watt) during the rise phase (defined as vertical velocity > 0.1m/s) of each single step were calculated. The highest mean power output was used in the analyses. For more detailed information on the calculation of power by means of 3D accelerometry, see previous work [28].

The 6MWT was performed once and all other functional tests twice. The best result was used in the analyses.

### Statistical analyses

One-way analysis of variance with Bonferroni post hoc testing was used to test for baseline differences and for differences in exercise adherence between groups. Non-parametric statistics were used for the questionnaire variables (ordinal scales). Between-group differences on the acceptability questionnaire were assessed with Mann-Whitney U (two groups) or Kruskal-Wallis tests (three groups) at all points in time. Time-effects were assessed with Friedman tests. Fisher’s exact test was used to check for differences in the number of drop-outs between groups.

To assess between-group differences in changes over time for the performance variables, linear mixed-model analysis with an unstructured covariance structure was used, with time as repeated factor and group as fixed factor. Post hoc analyses were conducted for within-group changes and to determine which groups differed in changes. Because of the risk for type II errors, these post hoc analyses were performed when the time effect or the time by group interaction effect showed at least a trend (p < 0.1) towards significance. In order to test the normality assumption for multilevel regression models, we checked for all models whether the residuals were normally distributed by means of Shapiro-Wilk tests. If a dependent variable was non-normally distributed, a log or square-root transformation was conducted. Only when these transformations did not result in normality, non-parametric tests were used as alternative. In that case, time effects from baseline to post intervention were analyzed with Friedman tests, and within-group changes from baseline to post were analyzed with Wilcoxon-signed rank tests. Percent changes from baseline to post were calculated and then used in Kruskal–Wallis tests to determine differences in changes between groups, with Mann-Whitney U tests as post hoc tests. The following parameters were not normally distributed: SA duration and power (non-parametric), STS duration (log transformation), SJ jump height and contraction time (non-parametric), CMJ concentric time (log transformation), DJ RPD (log transformation).

Cohen’s *d* effect sizes for between-group differences in percent changes from baseline to post-intervention were calculated. This was done for the variables that showed a significant difference in change between groups. Thresholds 0.20, 0.50 and 0.80 were used to interpret small, medium and large effect sizes [29].

All statistical tests were executed with SPSS software version 25 (SPSS Inc., Chicago, IL). Level of significance was set at p < 0.05.

## Results

Table 3 shows the baseline characteristics of the subjects in each group.

**Table 3:** Means ± SD for baseline characteristics of the subjects.

Characteristic	RT (N = 12)	PLYO (N = 14)	WALK (N = 14)	p-value
Age (years)	68.2 ± 2.7	69.6 ± 3.3	70.5 ± 5.1	0.334
Body height (cm)	170.1 ± 5.4	175.6 ± 5.8	177.6 ± 6.5*	0.008
Body mass (kg)	81.1 ± 9.9	86.1 ± 11.8	80.2 ± 10.2	0.305
BMI (kg/m^2^)	28.0 ± 2.8	27.9 ± 3.5	25.4 ± 2.8	0.060

RT = resistance training; PLYO = plyometric training; WALK = walking.

p-values: results of one-way analysis of variance.

*Significant difference with RT (p < 0.05).

### Feasibility

In total, 101 older men were assessed for eligibility. Twenty-nine men declined to participate and thirty-two were excluded (for reasons, see flowchart in Fig 1). Recruitment rate was 39.6%.

Of the 40 subjects that started the study, three in PLYO (one in week 5, two in week 6) and one in RT (in week 11) dropped out. The number of drop-outs was not different between groups (p = 0.190). Reasons for drop-out were knee pain (PLYO, n = 1), muscle strain in the m. gastrocnemius during the forward or sideways step-up exercise (PLYO, n = 2) and lower back pain (RT, n = 1). Other minor adverse effects included knee pain (n = 4 PLYO, n = 1 RT), mild muscle soreness (n = 5 PLYO, n = 4 RT), pain in glutes (n = 1 RT, n = 1 WALK), pain in feet (n = 1 WALK). Three subjects in PLYO, six in RT and twelve in WALK did not report any side effects over the 12-week period.

Exercise session adherence was higher in WALK (106.7 ± 18.6%) than in PLYO (79.8 ± 23.3%, p = 0.001) and RT (91.9 ± 8.4%, p = 0.137). However, when the adherence rate in WALK was corrected by excluding any exercise sessions above the weekly prescribed number, adherence in WALK dropped to 93.1 ± 7.8 and was no longer different from PLYO or WALK (p = 0.052). In addition, when subjects who dropped out were deleted from the analysis, adherence increased to 91.2 ± 4.4% in PLYO and 93.9 ± 4.8% in RT. The number of on-site group walks attended was 12.4 ± 9.6 (ranging from 1 to 29), which corresponds to once per week on average. Group size during these walks was 4 ± 2 subjects.

Acceptability of the exercise program was very high in both groups, with no difference between groups and no change over time (all p > 0.05). All subjects indicated that they were likely to engage in similar exercise programs in the future, apart from two subjects in RT, who gave a neutral answer (5/10) (Table 4).

**Table 4:** Means ± SD for questionnaire variables on acceptability of the exercise program.

Variables	RT	PLYO	WALK	p-value
Acceptability of the program				
Week 2	8.2 ± 1.1	8.5 ± 0.7		0.379
Week 4	8.1 ± 1.3	8.2 ± 1.0		0.935
Week 6	8.2 ± 1.3	8.3 ± 1.0		0.853
Week 8	8.2 ± 1.4	8.6 ± 0.6		0.510
Week 10	8.3 ± 1.1	8.8 ± 0.6		0.289
Week 12	8.4 ± 0.8	8.5 ± 0.6	8.6 ± 1.0	0.729
Likelihood of participation in similar exercise programs in the future				
Week 12	8.0 ± 1.8	9.1 ± 1.0	8.8 ± 1.7	0.305

RT = resistance training; PLYO = plyometric training; WALK = walking.

p-values: results of Mann-Whitney U tests (two groups) or Kruskal-Wallis tests (three groups).

### Performance outcomes

Analyses on baseline differences revealed that PLYO showed higher STS power than RT, PLYO produced more concentric power in SJ and CMJ than WALK, and RT had shorter concentric times during CMJ than WALK. Given that PLYO improved most on power production (see later) and that the comparison of RT and WALK was not the focus of this research, no corrections for these baseline differences were conducted.

Jump data from one subject in PLYO were deleted due to not being able to perform the tests in the correct manner and all performance data from one in WALK due to the influence of illness during testing.

#### 1-RM, force production and jump performance

**Fig 4:**
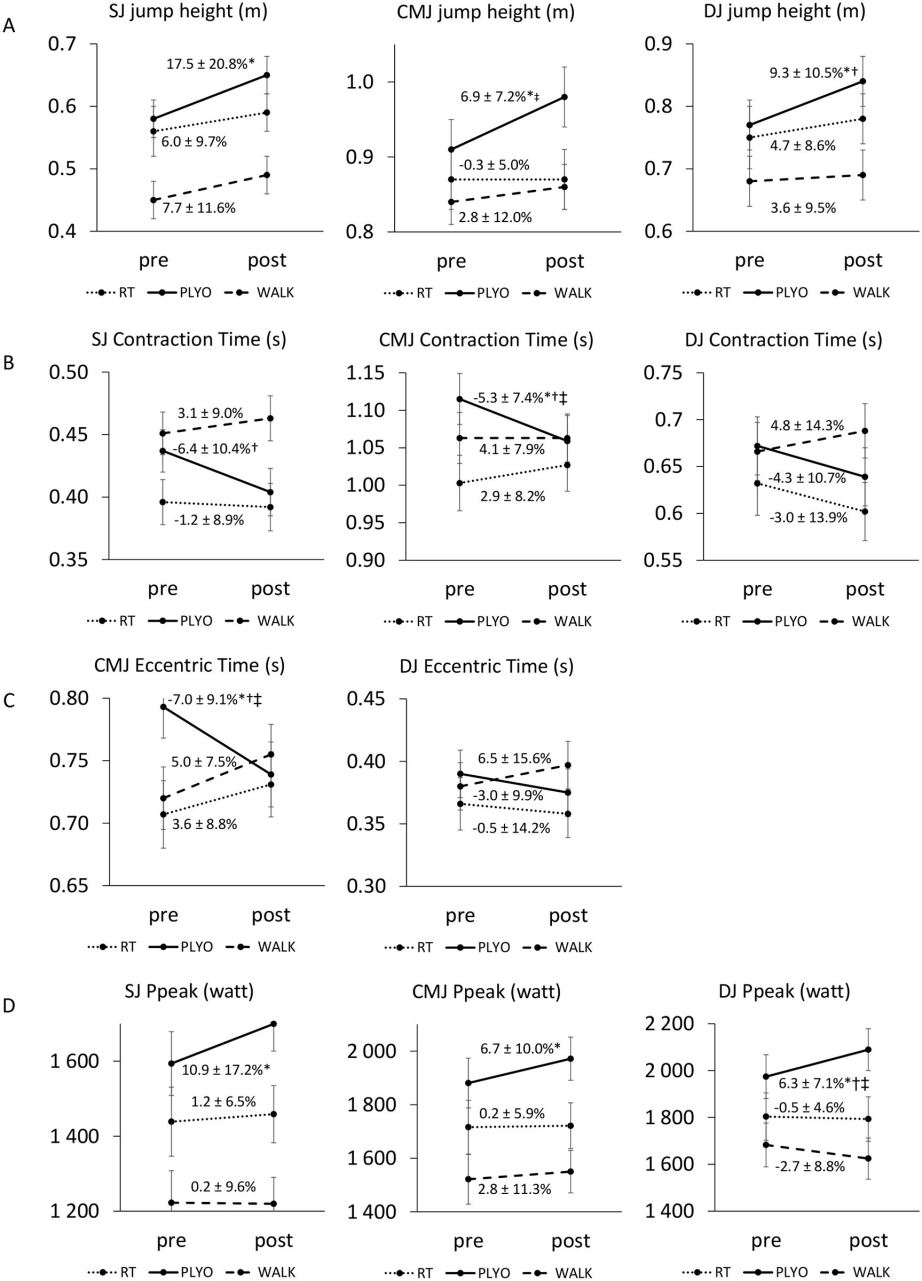
Estimated means and SE for jump height (A), contraction time (B), eccentric time (C), concentric peak power (Ppeak) (D) at baseline and post 12-weeks of plyometric training (PLYO), resistance training (RT) and walking (WALK) in squat jump (SJ), countermovement jump (CMJ) and drop jump (DJ). *Significant change from pre to post (p < 0.05); †Significant difference with WALK (p < 0.05); ‡ Significant difference with RT (p < 0.05).

Leg press 1-RM improved similarly in RT and PLYO and significantly more than in WALK (p < 0.001, *d* = 1.84 (RT vs WALK) and *d* = 1.47 (PLYO vs WALK)) (Table 5). The explosive isometric leg-extensor test did not show any time-by-group interaction effects. However, RT was the only group that increased maximal force (p = 0.028). There was no within-group change for RFD (Table 5). PLYO improved the most on jump performance. For an overview of the results, see S3–S5 Tables. With regard to SJ, PLYO was the only group that improved jump height (p = 0.017) (Fig 4A), reduced contraction time (p = 0.059) and exerted more power (both Ppeak and RPD) post-intervention (p < 0.05) (Fig 4B–4D). This improvement in contraction time and RPD was significantly different from WALK (p = 0.026, *d* = 0.98 and p = 0.014, *d* = 1.06 respectively). Similar results were found for CMJ. In PLYO, overall contraction time reduced because of a reduction in eccentric time, jump height increased and more concentric power was produced (p < 0.05). Overall contraction time and eccentric time improved significantly more in PLYO than in both RT and WALK (all p < 0.05, *d* ranged from 1.05 to 1.44), while jump height improved significantly more in PLYO than in RT (p = 0.030, *d* = 1.16) (Fig 4A–4D). With regard to DJ, PLYO was the only group that significantly increased jump height (Fig 4A) and RSI, and these increases were greater than in WALK (p = 0.017, *d* = 0.57 and p = 0.006, *d* = 0.98 respectively). In addition, PLYO was able to produce more power (Ppeak) in the concentric phase post-intervention. This gain in Ppeak was significantly different from both RT (p = 0.014, *d* = 1.14) and WALK (p = 0.001, *d* = 1.13) (Fig 4D).

**Table 5:** Estimated means and SE at baseline (pre-) and posttest and % change (±SD) for leg press one repetition maximum, leg-extensor maximal isometric force and rate of force development in the three intervention groups.

		RT			PLYO			WALK			Statistics	
		Mean	SE	%	Mean	SE	%	Mean	SE	%	Time	Time x group
1-RM (kg)	Pre	194.2	14.6		175.7	13.6		162.1	13.6			
	Post	244.3	15.1	25.0 ± 10.0*†	211.8	14.2	23.0 ± 13.6*†	161.7	14.1	2.9 ± 13.7	**F (1, 31.1) = 72.0; p < 0.001**	**F (2, 31.1) = 20.5; p < 0.001**
MVC (N)	Pre	815.7	32.8		787.9	30.4		725.3	30.4			
	Post	862.1	31.7	6.8 ± 10.7*	807.3	30.2	2.9 ± 7.1	727.7	29.3	-0.2 ± 9.7	F (1, 32.9) = 4.0; p = 0.053	F (2, 32.9) = 1.3; p = 0.285
RFD0-100 (N/s)	Pre	2982.2	282.0		2638.7	261.1		2632.8	261.1			
	Post	2717.0	254.2	-6.1 ± 25.0	2887.0	245.7	4.3 ± 15.7	2507.0	234.6	-4.0 ± 24.5	F (1, 33.3) = 1.1; p = 0.301	F (2, 33.3) = 1.0; p = 0.374

Statistics of Linear Mixed Models analyses.

*Significant change from pre to post (p < 0.05);

^†^Significant difference with WALK (p < 0.05).

PLYO = plyometric training, RT = resistance training, WALK = walking, MVC = maximal voluntary contraction, RFD = rate of force development.

#### Functional performance

Both 10m fast walk and 6MWD improved similarly in all groups, while STS duration did not change. No time-by-group interaction effect was found for STS power (p = 0.405), although only RT (p = 0.018) and PLYO (p = 0.011) showed a within-group gain. Interestingly, stair-climbing performance, represented by stair ascent duration and power, improved significantly more in PLYO than in RT (p < 0.05, *d* = 1.12 and *d* = 1.07 respectively) (Table 6).

**Table 6:** Estimated means and SE at baseline (pre-) and posttest and % change (±SD) for functional performance in the three intervention groups.

		RT			PLYO			WALK			Statistics	
		Mean	SE	%	Mean	SE	%	Mean	SE	%	Time	Time x group
10m fast walk (s)	Pre	4.50	0.23		4.41	0.21		4.42	0.21			
	Post	4.23	0.21	-5.3 ± 9.2*	4.12	0.20	-5.5 ± 8.6*	4.12	0.19	-6.5 ± 7.2*	**F (1, 34.3) = 23.2; p < 0.001**	F (2, 34.3) = 0.02; p = 0.983
6MWD (m)	Pre	579.6	21.9		600.6	20.2		604.2	20.2			
	Post	618.9	23.9	6.7 ± 6.5*	635.0	22.4	6.1 ± 4.9*	631.2	22.1	4.4 ± 3.8*	**F (1, 32.0) = 44.2; p < 0.001**	F (2, 32.0) = 0.5; p = 0.596
SA duration (s)	Pre	1.64	0.13		1.95	0.12		1.96	0.12			
	Post	1.67	0.10	2.1 ± 9.5	1.71	0.09	-10.1 ± 12.1*‡	1.85	0.09	-4.8 ± 7.6*	**χ**^**2**^ **(1) = 4.2; p = 0.040** (np)	**χ**^**2**^ **(2) = 6.0; p = 0.049** (np)
SA power (watt)	Pre	846.4	66.7		854.9	61.8		753.4	61.8			
	Post	825.7	64.2	-1.5 ± 9.8	961.9	60.7	18.0 ± 23.8*‡	774.1	59.3	2.0 ± 15.0	χ^2^ (1) = 0.1; p = 0.739 (np)	χ^2^ (2) = 4.8; p = 0.089 (np)
STS duration (s)	Pre	8.44	0.42		8.06	0.41		9.25	0.38			
	Post	8.90	0.42	5.4 ± 10.8	8.42	0.40	4.8 ± 7.5	9.38	0.40	1.6 ± 12.6	**F (1, 31.9) = 4.6; p = 0.040**	F (1, 31.9) = 0.5; p = 0.601
STS power (watt)	Pre	291.7	18.7		370.2	17.3		316.7	17.3			
	Post	329.6	18.3	14.6 ± 19.1*	410.6	17.7	12.9 ± 9.2*	331.9	16.9	5.8 ± 16.3	**F (1, 32.2) = 13.3; p = 0.001**	F (1, 32.2) = 0.9; p = 0.405

Statistics of Linear Mixed Models analyses; stair ascent duration and power were not normally distributed and non-parametric (np) tests were performed and reported for these variables; STS duration was not normally distributed and log transformed for the analyses. For easier interpretation, non-transformed data means are reported for all variables.

*Significant change from pre to post (p < 0.05)

^‡^ Significant difference with RT (p < 0.05)

PLYO = plyometric training, RT = resistance training, WALK = walking, 6MWD = 6-minute walk distance, SA = stair ascent, STS = sit-to-stand.

## Discussion

This study developed and implemented a 12-week age-adapted and progressive plyometric exercise program for community-dwelling older men and compared its effects to traditional resistance training and walking. Primary outcomes were muscle strength, muscle power, jump performance and functional capacity. In addition, the feasibility of the plyometric training program was investigated. The results show that (1) PLYO is more effective than RT and WALK for improving muscle power (Ppeak and RPD) and jump performance (jump height and contraction time), (2) PLYO is equally effective compared to RT for improving muscle strength (1-RM), (3) all interventions equally improve walking performance, but PLYO seems favorable for stair climbing performance, and (4) older men seem to accept the PLYO program to a similar extent as either the RT or WALK program, although risk for injuries might be greater in PLYO.

In line with previous findings [24, 30, 31] and the principle of training specificity, plyometric exercise resulted in more optimal jump performance than traditional resistance training or walking. In all jumps, PLYO improved jump height post-intervention (6.9–17.5%) and this coincided with an increase in concentric power production (both Ppeak and RPD) and a decrease in (eccentric) contraction time. In other words, PLYO was able to jump higher and more powerfully, while needing less time to jump. We should acknowledge that ICC appeared poor for the duration of the eccentric phase in the countermovement jump (0.48), even though CV% was good (9.7%). However, we should keep in mind that our reliability values are based on a comparison between familiarization and baseline measurements. In subjects that are new to jump tests, we can expect an improvement in jump strategy, i.e. a faster eccentric time and/or faster transition from eccentric to concentric movement, from familiarization to baseline. The ICC quantifies the between-subject variance in relation to the total variance, which also contains the variance within subjects [25]. As the variance within subjects will increase in case of a learning effect from familiarization to baseline, a lower ICC value will be obtained, especially when between-subject differences in means are small. This result should therefore be interpreted with caution and replication in future studies is warranted.

Although we can only speculate on the underlying mechanisms behind improved jumping performance, Hoffren-Mikkola et al. [30] demonstrated that improvements in jumping performance after plyometric exercise were achieved with shorter operating lengths of the m. gastrocnemius and, therefore, increased fascicle stiffness and improved tendon utilization [30]. In addition, Piirainen et al. [24] reported no increase in muscle activity of the triceps surae during explosive isometric tests after plyometric exercise, suggesting that mechanisms other than improved voluntary drive, such as increased utilization of elastic energy and/or stretch reflex activity, may be responsible for enhanced jump performance [24]. With regard to muscle architectural changes, 6 weeks of plyometric training has been shown to result in increased muscle thickness, fascicle length and pennation angle, which likely contributed to the observed changes in power [32].

In the current study, we did not observe a significant increase in leg-extensor RFD after either plyometric or resistance exercise. Such null findings should be set in context with respect to the body position specificity of the test and training exercises: a significant increase in RFD may be more likely if the test is more body position-specific or if training is performed with the intent to rapidly contract muscles [33]. Although RT included dynamic leg press training and a multi-joint isometric leg press set-up was used to assess RFD, training was performed bilaterally while testing unilaterally. In contrast, part of the training in PLYO was performed unilaterally, but in different body positions regarding knee-joint angle. We should however note that the percent change does seem to point out an exercise-induced improvement in PLYO, but this improvement was not significant because of a large variability in training responses and small sample size. Coefficient of variation (18.9%) was high, but that is quite typical in early phase RFD, even in young adults [34]. A longer time interval of 0–200 ms was considered (CV of 9.9%), but as it did not lead to any different conclusions, we decided not to include this information in the results section for brevity. In line with the results on RFD, no time-by-group interaction effect was found for isometric MVC, although RT was the only group that improved.

To our knowledge, limited reports exist on changes in 1-RM after plyometric exercise in older adults. Bolam et al. (2016) reported no increase in leg press 1-RM after 9 months of plyometric exercises performed at high (40–80 jumps) or moderate (20–40 jumps) dose 4x/weekly in middle-aged and older men. However, these findings might have been related to the limited adherence rates (i.e. 53–65%) [35]. On the contrary, Correa et al. [36] reported similar gains in knee-extension 1-RM after 6 weeks of plyometric compared to traditional resistance exercise. Both groups followed the same 6-week program of generalized strength training prior to division in two exercise groups. Gains in 1-RM from week 6 to 12 were similar (+20–21%) between groups [36]. This is in line with our findings, showing that short-term plyometric exercise is able to induce similar gains in leg press 1-RM as traditional resistance exercise. This gain in 1-RM might be linked to muscle architectural changes (i.e. increased muscle thickness and pennation angle) and improved muscle recruitment, as found previously after 6 weeks of plyometric training in older men [32]. Although the long-term effects should be investigated, these results show that PLYO is beneficial over RT for improving muscle power and jump performance without compromising gains in strength, at least when older individuals begin a high-intensity (resistance or plyometric) training program.

Improved (rapid) power production in PLYO was hypothesized to result in a greater gain in functional capacity compared to RT and WALK [12, 13, 36]. However, this hypothesis was only partly confirmed. Walking performance (10m fast walk and 6MWD) equally improved in RT, PLYO and WALK, and to a similar extent (4.4–6.7%) as previously reported after resistance exercise [22] or walking [20]. 5xSTS duration did not change significantly in either of the groups. This test was probably not challenging or training-specific (i.e. no SSC) enough in our sample of older men, as all subjects were classified as not mobility-limited (6MWD > 400m). Although 5xSTS duration did not change, the highest mean power output in a single sit-to-stand transition improved in both RT and PLYO. This result was not influenced by gains in body mass, as there was no correlation between gains in power and in body mass nor a significant gain in body mass post-intervention. Interestingly, PLYO showed a greater improvement in stair climbing performance than RT. This result was not surprising, given that PLYO improved power production in the leg-extensor (i.e. knee- and hip-extensor) muscles, which play a dominant role in developing the power needed to progress from one step to the next during stair ascent [37, 38]. In addition, the forward step-up (jump) exercises in PLYO are mechanically very similar to stair climbing, enhancing the potential of inducing training-specific adaptations. As stair climbing is one of the most demanding functional tasks in older adults, improvements are crucial in maintaining independence.

It should be noted that some of our key findings of between-group differences (i.e. results on stair ascent and squat jump) are based on non-parametric analyses. However, in most cases, non-parametric tests are considered to have lesser statistical power than parametric analyses, meaning that the latter are more likely to detect an effect when it actually exists. Parametric analyses of our non-normally distributed parameters resulted in the same conclusions, which indicates towards the robustness of our results.

A secondary aim of this study was to assess the feasibility of plyometric exercise in healthy older men. Recruitment rate was 39.6%, which is acceptable and in line with recruitment rates of (resistance) exercise trials in similar populations [22, 23, 39]. Exercise adherence was very high (>80%) in all individuals, except in the four dropouts, independent of training group. Subjects in WALK appeared to have higher adherence rates than the other groups, with some individuals even exceeding the recommended amount of training sessions. This might have been caused by the self-report in that group, with subjects noting down every daily life walk instead of solely the walks as part of the training program. When adherence rates in WALK were corrected by excluding any sessions above the prescribed training frequency, adherence was similar in all groups.

Acceptability of the exercise program was very high in both PLYO and RT, demonstrating that plyometric exercise is at least perceived by healthy older men as being feasible (and to a similar extent as resistance exercise). All subjects in PLYO indicated that they were likely to participate in similar exercise programs in the future. However, as shown previously, this intention to participate in future programs appears to have limited predictive value for actual long-term exercise behavior [40]. Also, inevitable in research with volunteers, a self-selection bias might have occurred, by only including highly motivated subjects in the intervention. In addition, subjects might have tended to give socially desirable answers to the questionnaires. Acceptability should therefore be investigated in larger study samples to confirm these findings.

Although our subjects seemed to enjoy the plyometric exercise program and considered it to be feasible, we cannot ignore the adverse events. Two in particular need further attention: knee pain and muscle strain of the m. gastrocnemius. Five out of 14 subjects in PLYO reported some kind of knee pain during the exercise program. In one subject, this was related to a non-treated knee injury in the past and not specifically caused by the training program. In three subjects, knee pain was only reported once in the beginning weeks of the exercise program and disappeared later on. This might have been related to the height of the box (up to 30–40 cm) during step-up exercises, which may have caused an unfavorable knee joint angle (more flexed), or poor technique (e.g. inversion at the knee, corrected when inspected by supervisors). Height of the box was reduced once subjects progressed to jumping (20–30 cm) and knee pain was no longer reported. In only one subject, knee pain was sufficiently severe to cause a drop-out. It is not clear whether this pain was caused by the stepping exercises as such or by the impact of the landing phase during jumping. A more severe adverse event, causing two subjects to drop out, was a strain in the m. gastrocnemius. In both cases, the injury occurred in the second phase of the plyometric program (week 5 or 6), in which slight, sub-maximal jumps were performed to familiarize with the exercises. The injuries did not seem to be caused by insufficient warm-up, nor by excessive fatigue, as they occurred in set 3 or set 6 (out of the total exercise volume of 9 pre-programmed sets). Importantly, the strain occurred during the concentric phase of either the forward or the sideways step-up jump. These are both unilateral exercises in which the calf muscles are part of the prime movers. Considering that both subjects could be classified as obese (BMI of 30.5 and 33.45 kg/m^2^ respectively), the calf muscles had to comply with high absolute loading forces. Excessive body weight in combination with a likely decrease in gastrocnemius fascicle length [15] and Achilles tendon stiffness [15, 41] with ageing inevitably increases the risk of muscle injuries during such exercises.

With this in mind, we should reflect on the proper design and exercises to be included in a plyometric exercise program for older adults. To be able to compare the effects of a plyometric-only protocol to traditional resistance exercise, we did not include a preparation phase of resistance training before introducing jumps. It can be argued that a proper periodization design is warranted, starting with traditional resistance exercise aimed to induce hypertrophy and maximal strength gains, before progressing to explosive-type of exercises. However, we did progress slowly from slow-speed exercises without jumping over submaximal jumps to maximal jumps. The three plyometric exercises are all considered to be low-intensity drills [42]. Countermovement jumps have been used multiple times in exercise programs for healthy older adults without any adverse events [43, 44]. Step-up exercises were chosen considering their beneficial effects on muscle strength and functional performance [23] and their resemblance to stair climbing, i.e. one of the most demanding functional tasks for older adults. In addition, the lateral step-up jumps were already used in older women without any adverse events [36]. Subjects in our study were recommended to use wall bars if necessary (both to reduce intensity and the balance component), but only one subject chose to do so. Training volume per session, in plyometrics typically expressed as total number of foot contacts, was limited to recommended guidelines for beginners (i.e. 80 to 100) [42] and even decreased with increasing training intensity to keep injury risk to the minimum. Adequate recovery, i.e. between repetitions (5 s, consecutive jumps only in last training phase), between sets (1 min.), between exercises (at least 2 min.) and between training sessions (48 – 72h), was provided [42]. Although we did not measure markers of muscle damage, subjects in PLYO did not report excessive muscle soreness after training. However, given that plyometric exercise interventions in older adults remain scarce in literature, more research is urgently needed to set the appropriate training dose (volume, intensity, frequency, duration) [18] and exercises for optimizing gains in power and functional capacity and for minimizing injury risk. In addition, we should acknowledge that it is challenging to quantify exercise intensity (i.e. both mechanical load and neuromuscular demand) [45] and fatigue during plyometric exercises, although it would definitely be of added value to optimize training dose.

Based on the findings of the current study, people might question whether it really is worth doing more stressful plyometric jump training as opposed to regular walking. Aside from training-specific gains in strength, power, jump and stair climbing performance, more ‘neutral’ functional performance tests (i.e. walking and STS) do not seem to improve as robustly. However, the following aspects need to be considered. Firstly, it should be noted that the relationship between muscle power and functional performance is curvilinear [5]. Hence, for low levels of muscle power, the improvement in power leads to a substantial improvement in functional performance. However, above a certain level of baseline muscle power, further increases in power do not lead to further increases in the parameters usually assessed to register functional performance (e.g. STS ability). Our subjects were well-functioning older men with relatively high baseline levels of muscle power, which might explain the null findings with regard to STS ability. Notwithstanding, any improvement in muscle power in well-functioning older adults should be recognized as important, even if it does not result in further improvements in functional performance. Higher levels of muscle power can at least postpone the drop below the disability threshold. Secondly, STS ability is subject to a ceiling effect in well-functioning adults, as noted previously [28]. Instead of questioning the beneficial effect of plyometric exercise on functional performance, it may be necessary to question whether traditional functional performance tests are sensitive enough to capture changes in well-functioning older adults. Jumping is a more sensitive measure of power performance than chair rising [46]. Even though jumping is not an activity that older adults do on a regular basis, the inability to jump is related to poorer self-reported health, more comorbidities, worse cognitive functioning, more limitations in daily life activities and higher fall incidence [47]. Thirdly, intensity is not something to be feared, as discussed by Hunter et al. [48] and commented by Gentil et al. [49]. Ageing is associated with both a decline in type II fiber size and in the ability to activate these fibers [50, 51], resulting in decreased strength and power production. To train these type II fibers, high efforts are needed, either through using relatively high loads (as in RT), performing exercises at high velocity (as in PLYO) or training to momentary failure [49]. According to Henneman’s size principle, walking at comfortable pace is not sufficient to target the type II fibers and is therefore incapable of countering this age-related decline.

Considering the cost-benefit of our plyometric exercise program, we cannot claim that plyometric training is better than concentric-only machine-based power training, as previously performed by several research groups [13, 52]. While concentric-only machine-based power training or alternative plyometric training with machines designed to limit the impact of the landing phase (e.g. [24, 32]) might be considered a safer modality for older adults, adverse events in the current study did not seem to be the result of high impact during landing. Because of differences in the design of the traditional resistance exercise protocol as reference, in the study population and in measurement outcomes, it is also difficult to compare our results to previous findings in machine-based power training [13, 52]. What we can say is that both plyometric and machine-based power training will improve power and functional performance in older adults previously unaccustomed to systematic training, although the mechanisms behind these improvements are very likely training-mode specific. While neuromuscular improvements due to machine-based power training are mainly attributable to improved voluntary neural drive, plyometric training seems to result in a better utilization of the advantaged provided by the SCC [24]. Both aspects are vulnerable to age-related deterioration [15, 16, 51] and deserve attention in exercise programs for older adults.

To conclude, plyometric exercises are beneficial over traditional resistance training for improving muscle power, jump and stair climbing performance without compromising gains in muscle strength. This form of training seems feasible in older men, although proper supervision is warranted and caution is advised when applying unilateral exercise drills because of a potential increase in the risk for calf muscle injuries. Box heights of 20–30 cm are feasible for step-up jumps in older men, but higher heights might result in more reports of knee pain because of unfavorable knee-joint angles. Given the beneficial performance-related effects of plyometric exercise in older adults, future research should focus on optimizing the training dose, exercise drills and periodization schemes.

## Supporting information

S1 Table Reliability values for jump parameters by comparing familiarization and baseline measurements. (DOC)

S2 Table Reliability values for the explosive isometric leg-extensor test by comparing familiarization and baseline measurements. (DOC)

S3 Table Estimated means and SE at baseline (pre-) and post-intervention and % change (±SD) for squat jump in the three intervention groups. (DOC)

S4 Table Estimated means and SE at baseline (pre-) and posttest and % change (±SD) for countermovement jump (CMJ) in the three intervention groups. (DOC)

S5 Table Estimated means and SE at baseline (pre-) and posttest and % change (±SD) for drop jump in the three intervention groups. (DOC)
