# Supplementary material for: An age-adapted plyometric exercise program improves dynamic strength, jump performance and functional capacity in older men either similarly or more than traditional resistance training
Source: PLoS One. 2020 Aug 25;15(8):e0237921. doi: 10.1371/journal.pone.0237921 (PMC7447006; doi:10.1371/journal.pone.0237921)
Supplement: S1 Table — (DOC) [file pone.0237921.s001.doc]

**S1 Table.** Reliability values for jump parameters by comparing familiarization and baseline measurements.

|  | Squat jump | | | Countermovement jump | | | Drop jump | | |
| --- | --- | --- | --- | --- | --- | --- | --- | --- | --- |
|  | ICC(3,1) | CV(%) | TEM(%) | ICC(3,1) | CV(%) | TEM(%) | ICC(3,1) | CV(%) | TEM(%) |
| Contraction time (s) | 0.59 | 10.5 | 10.5 | 0.63 | 7.2 | 7.6 | 0.74 | 9.3 | 9.2 |
| Jump height (m) | 0.84 | 9.9 | 10.6 | 0.80 | 8.0 | 8.7 | 0.93 | 6.2 | 6.3 |
| RSI (mm/s) |  |  |  |  |  |  | 0.89 | 10.3 | 10.2 |
| ***Eccentric*** |  |  |  |  |  |  |  |  |  |
| Ecc Time (s) |  |  |  | 0.48 | 9.7 | 10.2 | 0.70 | 10.9 | 10.8 |
| ***Concentric*** |  |  |  |  |  |  |  |  |  |
| Ppeak (watt) | 0.80 | 11.1 | 11.4 | 0.81 | 8.8 | 10.0 | 0.90 | 6.6 | 6.7 |
| RPD (watt/s) | 0.73 | 17.5 | 17.4 | 0.68 | 18.2 | 18.9 | 0.80 | 14.5 | 14.4 |
| Conc Time (s) | 0.59 | 10.5 | 10.5 | 0.84 | 5.9 | 6.0 | 0.75 | 9.1 | 9.0 |

Ppeak = peak power, RPD = rate of power development, RSI = reactive strength index, ICC = intraclass correlation coefficient, CV = coefficient of variation, TEM = technical error of measurement
