# Supplementary material for: An age-adapted plyometric exercise program improves dynamic strength, jump performance and functional capacity in older men either similarly or more than traditional resistance training
Source: PLoS One. 2020 Aug 25;15(8):e0237921. doi: 10.1371/journal.pone.0237921 (PMC7447006; doi:10.1371/journal.pone.0237921)
Supplement: S2 Table — (DOC) [file pone.0237921.s002.doc]

**S2 Table.** Reliability values for the explosive isometric leg-extensor test by comparing familiarization and baseline measurements.

|  | ICC(3,1) | CV(%) | TEM(%) |
| --- | --- | --- | --- |
| MVC (N) | 0.88 | 5.7 | 5.7 |
| RFD0-100 (N/s) | 0.78 | 18.9 | 18.8 |
| RFD0-200 (N/s) | 0.83 | 9.9 | 10.1 |

MVC = maximal voluntary contraction, RFD = rate of force development, ICC = intraclass correlation coefficient, CV = coefficient of variation, TEM = technical error of measurement
