# Supplementary material for: An age-adapted plyometric exercise program improves dynamic strength, jump performance and functional capacity in older men either similarly or more than traditional resistance training
Source: PLoS One. 2020 Aug 25;15(8):e0237921. doi: 10.1371/journal.pone.0237921 (PMC7447006; doi:10.1371/journal.pone.0237921)
Supplement: S3 Table — (DOC) [file pone.0237921.s003.doc]

**S3 Table.** Estimated means and SE at baseline (pre-) and post-intervention and % change (±SD) for squat jump in the three intervention groups.

|  |  | RT | | | PLYO | | | WALK | | | statistics | |
| --- | --- | --- | --- | --- | --- | --- | --- | --- | --- | --- | --- | --- |
|  |  | Mean | SE | % | Mean | SE | % | Mean | SE | % | Time | Time x group |
| Contraction time (s) | Pre | 0.396 | 0.018 |  | 0.437 | 0.017 |  | 0.451 | 0.017 |  |  |  |
|  | Post | 0.392 | 0.019 | -1.2 ± 8.9 | 0.404 | 0.019 | -6.4 ± 10.4† | 0.463 | 0.018 | 3.1 ± 9.0 | χ² (1) = 1.5; p = 0.223 (np) | χ² (2) = 5.1; p = 0.077 (np) |
| Jump height (m) | Pre | 0.56 | 0.04 |  | 0.58 | 0.03 |  | 0.45 | 0.03 |  |  |  |
|  | Post | 0.59 | 0.03 | 6.0 ± 9.7 | 0.65 | 0.03 | 17.5 ± 20.8* | 0.49 | 0.03 | 7.7 ± 11.6 | **χ² (1) = 11.8; p = 0.001** (np) | χ² (2) = 1.9; p = 0.388 (np) |
| Ppeak(watt) | Pre | 1439 | 92 |  | 1594 | 85 |  | 1223 | 85 |  |  |  |
|  | Post | 1459 | 76 | 1.2 ± 6.5 | 1699 | 73 | 10.9 ± 17.2* | 1220 | 71 | 0.2 ± 9.6 | F (1, 32.7) = 3.2; p = 0.085 | F (2, 32.7) = 2.0; p = 0.147 |
| RPD (watt/s) | Pre | 4579 | 342 |  | 4592 | 317 |  | 3577 | 317 |  |  |  |
|  | Post | 4796 | 321 | 4.4 ± 14.3 | 5228 | 311 | 20.7 ± 27.6*† | 3464 | 297 | -2.9 ± 15.3 | **F (1, 31.5) = 4.3; p = 0.047** | **F (2, 31.5) = 3.3; p = 0.048** |

statistics of Linear Mixed Models analyses; jump height and contraction time were not normally distributed and non-parametric tests were performed and reported for these variables. For easier interpretation, means are reported for all variables.

PLYO = plyometric training, RT = resistance training, WALK = walking, Ppeak = peak power, RPD = rate of power development

*Significant change from pre to post (p < 0.05); †Significant difference with WALK (p < 0.05)
