# Supplementary material for: An age-adapted plyometric exercise program improves dynamic strength, jump performance and functional capacity in older men either similarly or more than traditional resistance training
Source: PLoS One. 2020 Aug 25;15(8):e0237921. doi: 10.1371/journal.pone.0237921 (PMC7447006; doi:10.1371/journal.pone.0237921)
Supplement: S4 Table — (DOC) [file pone.0237921.s004.doc]

**S4 Table.** Estimated means and SE at baseline (pre-) and posttest and % change (±SD) for countermovement jump (CMJ) in the three intervention groups.

|  |  | RT | | | PLYO | | | WALK | | | statistics | |
| --- | --- | --- | --- | --- | --- | --- | --- | --- | --- | --- | --- | --- |
|  |  | Mean | SE | % | Mean | SE | % | Mean | SE | % | Time | Time x group |
| Contraction time (s) | Pre | 1.003 | 0.037 |  | 1.115 | 0.034 |  | 1.063 | 0.034 |  |  |  |
|  | Post | 1.027 | 0.035 | 2.9 ± 8.2 | 1.059 | 0.034 | -5.3 ± 7.4*†‡ | 1.063 | 0.032 | 4.1 ± 7.9 | F (1, 32.8) = 0.04; p = 0.840 | **F (2, 32.8) = 4.0; p = 0.027** |
| Jump height (m) | Pre | 0.87 | 0.04 |  | 0.91 | 0.04 |  | 0.84 | 0.04 |  |  |  |
|  | Post | 0.87 | 0.04 | -0.3 ± 5.0 | 0.98 | 0.04 | 6.9 ± 7.2*‡ | 0.86 | 0.03 | 2.8 ± 12.0 | **F (1, 32.8) = 4.8; p = 0.035** | F (1, 32.8) = 2.7, p = 0.082 |
| ***Eccentric*** |  |  |  |  |  |  |  |  |  |  |  |  |
| Ecc Time (s) | Pre | 0.707 | 0.027 |  | 0.793 | 0.025 |  | 0.720 | 0.025 |  |  |  |
|  | Post | 0.731 | 0.026 | 3.6 ± 8.8 | 0.739 | 0.026 | -7.0 ± 9.1*†‡ | 0.755 | 0.024 | 5.0 ± 7.5 | F (1, 32.5) = 0.02; p = 0.892 | **F (2, 32.5) = 5.9; p =** **0.007** |
| ***Concentric*** |  |  |  |  |  |  |  |  |  |  |  |  |
| Ppeak (watt) | Pre | 1716 | 100 |  | 1881 | 93 |  | 1522 | 93 |  |  |  |
|  | Post | 1721 | 86 | 0.2 ± 5.9 | 1972 | 81 | 6.7 ± 10.0* | 1550 | 79 | 2.8 ± 11.3 | F (1, 33.4) = 3.7; p = 0.065 | F (2, 33.4) = 1.3; p = 0.284 |
| RPD (watt/s) | Pre | 9666 | 804 |  | 9686 | 744 |  | 7655 | 744 |  |  |  |
|  | Post | 10097 | 723 | 6.4 ± 21.3 | 10183 | 695 | 13.0 ± 20.5 | 7695 | 668 | 1.1 ± 15.2 | F (1, 32.6) = 1.6; p = 0.210 | F (2, 32.5) = 0.3; p = 0.716 |
| Conc Time (s) | Pre | 0.295 | 0.013 |  | 0.321 | 0.012 |  | 0.343 | 0.012 |  |  |  |
|  | Post | 0.296 | 0.011 | 1.1 ± 7.6 | 0.319 | 0.011 | -0.6 ± 7.0 | 0.349 | 0.010 | 2.1 ± 9.1 | F (1, 34.3) = 0.3; p = 0.582 | F (2, 34.3) = 0.2; p = 0.848 |

statistics of Linear Mixed Models analyses; concentric time was not normally distributed and log transformed for the analyses. For easier interpretation, non-transformed data means are reported.

PLYO = plyometric training, RT = resistance training, WALK = walking, Ppeak = peak power, RPD = rate of power development

*Significant change from pre to post (p < 0.05); †Significant difference with WALK (p < 0.05); ‡ Significant difference with RT (p < 0.05)
