# Supplementary material for: An age-adapted plyometric exercise program improves dynamic strength, jump performance and functional capacity in older men either similarly or more than traditional resistance training
Source: PLoS One. 2020 Aug 25;15(8):e0237921. doi: 10.1371/journal.pone.0237921 (PMC7447006; doi:10.1371/journal.pone.0237921)
Supplement: S5 Table — (DOC) [file pone.0237921.s005.doc]

**S5 Table.** Estimated means and SE at baseline (pre-) and posttest and % change (±SD) for drop jump in the three intervention groups.

|  |  | RT | | | PLYO | | | WALK | | | statistics | |
| --- | --- | --- | --- | --- | --- | --- | --- | --- | --- | --- | --- | --- |
|  |  | Mean | SE | % | Mean | SE | % | Mean | SE | % | Time | Time x group |
| Contraction time (s) | Pre | 0.632 | 0.035 |  | 0.672 | 0.032 |  | 0.666 | 0.032 |  |  |  |
|  | Post | 0.602 | 0.033 | -3.0 ± 13.9 | 0.639 | 0.032 | -4.3 ± 10.7 | 0.688 | 0.030 | 4.8 ± 14.3 | F (1, 33.8) = 1.0; p = 0.322 | F (2, 33.7) = 1.7; p = 0.199 |
| Jump height (m) | Pre | 0.75 | 0.05 |  | 0.77 | 0.04 |  | 0.68 | 0.04 |  |  |  |
|  | Post | 0.78 | 0.04 | 4.7 ± 8.6 | 0.84 | 0.04 | 9.3 ± 10.5*† | 0.69 | 0.04 | 3.6 ± 9.5 | **F (1, 32.4) = 16.0; p < 0.001** | F (2, 32.3) = 3.3; p = 0.052 |
| RSI (mm/s) | Pre | 1221.2 | 94.7 |  | 1172.0 | 87.7 |  | 1055.8 | 87.7 |  |  |  |
|  | Post | 1324.1 | 98.2 | 9.0 ± 11.7* | 1340.8 | 93.4 | 15.4 ± 15.4*† | 1042.9 | 90.8 | 0.4 ± 15.2 | **F (1, 31.3) = 11.7; p = 0.002** | **F (2, 31.3) = 4.6; p = 0.018** |
| ***Eccentric*** |  |  |  |  |  |  |  |  |  |  |  |  |
| Ecc Time (s) | Pre | 0.366 | 0.022 |  | 0.390 | 0.020 |  | 0.380 | 0.020 |  |  |  |
|  | Post | 0.358 | 0.020 | -0.5 ± 14.2 | 0.375 | 0.020 | -3.0 ± 9.9 | 0.397 | 0.019 | 6.5 ± 15.6 | F (1, 33.7) = 0.06; p = 0.803 | F (2, 33.7) = 1.4; p = 0.266 |
| ***Concentric*** |  |  |  |  |  |  |  |  |  |  |  |  |
| Ppeak (watt) | Pre | 1803.5 | 105.0 |  | 1974.0 | 97.2 |  | 1682.5 | 97.2.5 |  |  |  |
|  | Post | 1793.0 | 98.8 | -0.5 ± 4.6 | 2089.1 | 92.7 | 6.3 ± 7.1*†‡ | 1624.4 | 91.4 | -2.7 ± 8.8 | F (1, 32.0) = 0.7; p = 0.424 | **F (2, 32.0) = 7.2; p = 0.003** |
| RPD (watt/s) | Pre | 10047.4 | 869.9 |  | 10109.2 | 805.4 |  | 8566.3 | 805.4 |  |  |  |
|  | Post | 10500.5 | 922.4 | 8.3 ± 20.6 | 11502.8 | 901.4 | 15.9 ± 23.7† | 8192.8 | 851.8 | -4.9 ± 16.5 | F (1, 31.4) = 1.6; p = 0.214 | F (2, 31.4) = 2.5; p = 0.096 |
| Conc Time (s) | Pre | 0.266 | 0.015 |  | 0.282 | 0.014 |  | 0.287 | 0.014 |  |  |  |
|  | Post | 0.244 | 0.014 | 1.1 ± 7.6 | 0.264 | 0.014 | -5.9 ± 14.3 | 0.291 | 0.013 | 3.1 ± 15.0 | F (1, 33.7) = 3.6; p = 0.066 | F (2, 33.7) = 1.8; p = 0.183 |

statistics of Linear Mixed Models analyses; RPD was not normally distributed and log transformed for the analyses. For easier interpretation, non-transformed data means are reported.

PLYO = plyometric training, RT = resistance training, WALK = walking, Ppeak = peak power, RPD = rate of power development, RSI = reactive strength index

*Significant change from pre to post (p < 0.05); †Significant difference with WALK (p < 0.05); ‡ Significant difference with RT (p < 0.05)
